# Carotid Space Mass Proximal to Vagus Nerve Causing Asystole and Syncope

**DOI:** 10.1155/2016/9306784

**Published:** 2016-07-19

**Authors:** Julie Leviter, Daniel H. Wiznia

**Affiliations:** Yale University School of Medicine, New Haven, CT 06511, USA

## Abstract

Manipulation of vagal nerve rootlets, whether surgical or through mass effect of a neoplasm, can result in asystole and hypotension, accompanied by ST depression and right bundle branch block. There are few case reports of a neoplasm causing these effects, and this case describes a patient with such a mass presenting with syncopal episodes. A 43-year-old man with a past medical history of HIV, bipolar disorder, and epilepsy was admitted to the neurology service for a video electroencephalogram (vEEG) to characterize syncopal episodes that were felt to be epileptic in origin. During the study, he experienced symptoms of his typical aura, which correlated with a transient symptomatic high degree AV block on telemetry, and an absence of epileptic findings on vEEG. Magnetic Resonance Imaging (MRI) of the brain showed a mass in the left posterior carotid space at the skull base. The patient underwent permanent dual chamber MRI-compatible pacemaker placement for his heart block. His syncopal episodes resolved, but presyncopal symptoms persisted. We discuss the presentation and treatment of vagal neoplasms.

## 1. Introduction

The vagus nerve provides parasympathetic innervation to the sinoatrial node, atria, and ventricles of the heart. Manipulation of vagal nerve rootlets, whether surgical or through mass effect of a neoplasm, can result in asystole and hypotension, accompanied by ST depression and right bundle branch block [1–3]. This can present clinically with syncope. We present the clinical findings in a patient with a left carotid space mass that was causing syncopal episodes through compression of the vagus nerve. These episodes were eliminated with a pacemaker, yet presyncopal symptoms persisted.

## 2. Case Presentation

A 43-year-old man with a past medical history of HIV, bipolar disorder, and epilepsy was admitted to the neurology service for a video electroencephalogram (vEEG) to characterize syncopal episodes that were felt to be epileptic in origin. These episodes started about ten years priorly and were associated with a preceding aura followed by loss of consciousness. He described this aura as a numbness that spread from head to toe and left him unresponsive for several minutes. He denied posturing, incontinence, eye deviation, tongue biting, or clonic activity during the events. He reported postevent disorientation and lethargy lasting from several hours to a full day. He had been on multiple antiepileptic medications without resolution of his episodes.

On examination, the patient was an anxious appearing well-built gentleman with normal vital signs and normal cardiac and neurologic exams.

**Figure 1:**
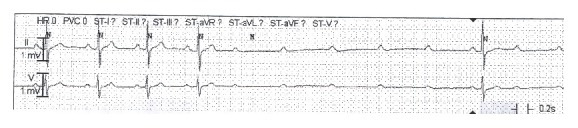
Cardiac telemetry recorded during patient's “aura” type symptoms demonstrating transient high degree AV block with pauses of up to six seconds.

**Figure 2:**
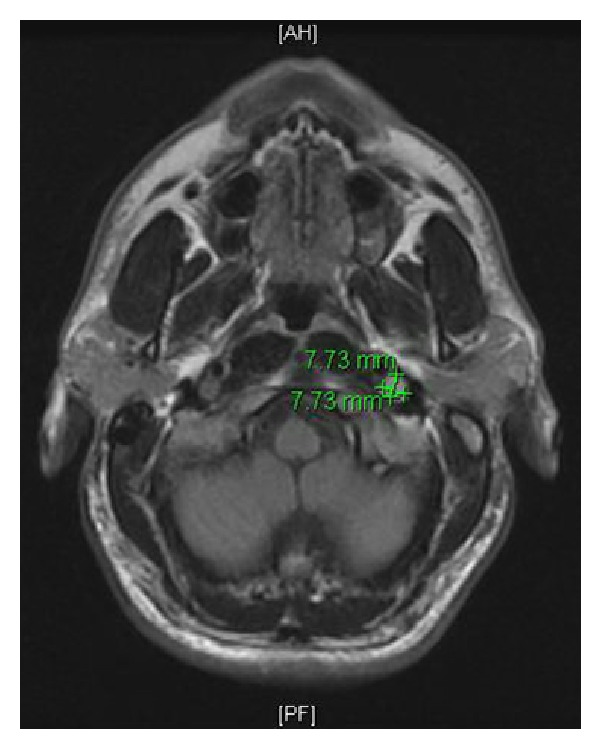
Axial Flair brain MRI demonstrating a mass in the left posterior carotid space at the skull base.

During the vEEG, he experienced symptoms of his typical aura. Concurrently, his vEEG activity was normal, but his cardiac telemetry demonstrated pauses of up to six seconds (Figure 1), reflecting a transient symptomatic high degree AV block. He was transferred to the cardiac care unit for close monitoring and temporary pacemaker placement. An MRI of the brain and neck showed a mass in the left posterior carotid space at the skull base (Figure 2). The differential of this mass included a left vagal schwannoma or a lymph node in proximity to the vagus nerve.

Neurosurgery declined surgical intervention, recommending Lamotrigine as seizure prophylaxis and serial MRIs to examine progression of the mass. The patient underwent permanent dual chamber MRI-compatible pacemaker placement for his heart block during the admission and was discharged with plans for outpatient follow-up.

Follow-up MRI after 4 months revealed a stable left carotid space mass. The patient denied further syncopal episodes since pacemaker implantation. He did, however, endorse episodes of numbness, decreased vision, and change in alertness occurring nearly daily, for which he was advised to consult his cardiologist.

## 3. Discussion

The vagus nerve provides parasympathetic innervation to the sinoatrial node, atria, and ventricles of the heart. It is also involved in the baroreceptor reflex, which responds to elevated blood pressure by increasing parasympathetic tone via decreased heart rate. Its path begins in the medulla oblongata, passes through the jugular foramen, and extends into the carotid sheath between the internal carotid artery and the internal jugular vein. The nerve then continues down to the neck, chest, and abdomen, where it helps to innervate the viscera. Cardiovascular effects of vagal disturbance similar to those seen in our patient have been described during surgical procedures involving manipulation of the vagal nerve rootlets [3, 4]. Nagashima et al. described a patient with asystole and hypotension, accompanied by ST depression and right bundle branch block, following intraoperative palpation of the vagus nerve.

Neoplasms of, or affecting, the vagus nerve include schwannomas, paragangliomas, branchial cleft cysts, malignant lymphomas, and metastatic cervical lymphadenopathy. Most schwannomas of the 9th, 10th, and 11th cranial nerves are slow-growing and histologically benign and do not invade surrounding structures. Their main effect is due to pressure exerted on the surrounding cranial nerves, cerebellum, and brainstem. If symptomatic, they commonly present with hoarseness, vertigo, tinnitus, cervical pain, or paroxysmal cough [5]. Vagal paragangliomas can have a similar clinical presentation [6].

Several case reports have described a presentation similar to that of our patient, in which a vagal neoplasm caused episodes of asystole and syncope [2, 6]. Okmen et al. present a case of a man with a vagal paraganglioma who presented with reflex cardiovascular syncope [6]. This patient was initially treated with a pacemaker, which reduced the frequency of attacks. However, he ultimately required surgical removal of the mass, which successfully resolved the episodes. In a similar case described by Sawamura and de Tribolet, a patient with a vagal schwannoma in the cerebellomedullary angle presented with syncopal attacks and postural hypotension. This patient's symptoms were relieved with surgical removal of the neoplasm [2].

Although rare, carotid mass should be included in the differential of a patient with syncopal or epileptic events. Imaging is central to the diagnosis of a vagal nerve neoplasm, with MRI being the study of choice [5]. This imaging modality can both reveal the diagnosis and provide important preoperative anatomic information about the neoplasm. Symptoms of cardiovascular reflex syncope can often be relieved with implantation of a defibrillator, but definitive treatment is complete surgical excision.
